# Methamphetamine-Induced Bowel Ischemia in a 50-Year-Old Male

**DOI:** 10.1155/2022/9690034

**Published:** 2022-04-05

**Authors:** Brian Kurtz, Abdalhai Alshoubi, Katrina Nguyen, Eric Gehres

**Affiliations:** St. Joseph's Medical Center/Dignity Health, 1800 N California St., Stockton, CA 95204, USA

## Abstract

Methamphetamine intoxication is a known risk factor for nonocclusive mesenteric ischemia (NOMI). We describe a case of a 50-year-old male with a history of polysubstance abuse who presented to the Emergency Department with severe abdominal pain and coffee-ground emesis. Computed tomographic (CT) imaging demonstrated portal venous gas and diffuse colonic wall thickening concerning for ischemic colitis. The patient underwent exploratory laparotomy with resection of the ascending colon as well as a necrotic section of the jejunum. Further embolic workup was negative with a subjective history of amphetamine use prior to presentation. NOMI has a high fatality rate, and we recommend providers include drug-induced bowel infarction on their differential when presented with findings of ischemic bowel of unclear etiology.

## 1. Introduction

Several case reports exist in the literature documenting an association between NOMI and methamphetamine (MA) use [1–9]. MA is a sympathomimetic with known vasospastic and vasoconstrictive properties that can compromise splanchnic perfusion and lead to intestinal ischemia [9]. The devastating cerebrovascular and cardiovascular health effects of methamphetamine abuse have been well-documented, but its impact on gastrointestinal pathology remains underappreciated.

## 2. Case Presentation

A 50-year-old gentleman with a history of treated hepatitis C infection presented to the Emergency Department accompanied by family with a two to three-day history of altered mental status, progressively worsening abdominal pain, coffee-ground emesis, and dark stools. Vital signs on arrival included a temperature of 36.6 degrees Celsius, a blood pressure of 128/60 mm Hg, a pulse rate of 173 bpm, and an oxygen saturation of 100% on room air. The patient became increasingly agitated and uncooperative with staff and was subsequently chemically sedated.

Pertinent laboratory findings included the following: white blood cell count 38.1 × 10,000/*μ*L (4,500-11,000/*μ*L), bicarbonate 15.0 mEq/L (23-30 mEq/L), anion gap 33 mEq/L (3-10 mEq/L), blood urea nitrogen 50.5 mg/dL (8-20 mg/dL), creatinine 5.3 mg/dL (0.74-1.35 mg/dL), lactic acid 6.0 mEq/L, (<2.3 mEq/L), and negative blood alcohol level.

**Figure 1:**
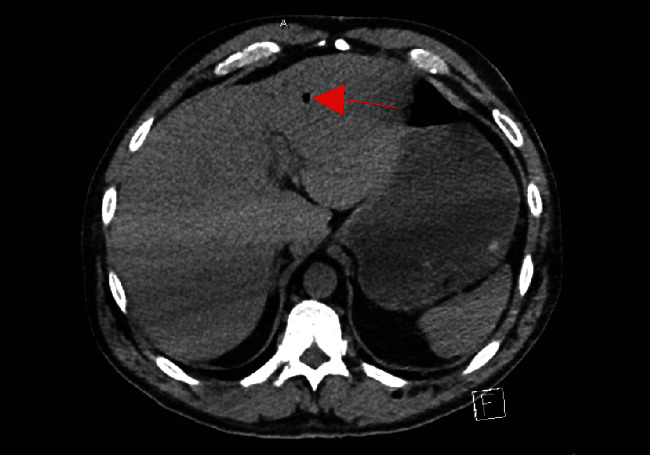
Noncontrast computed tomography with evidence of portal venous gas.

**Figure 2:**
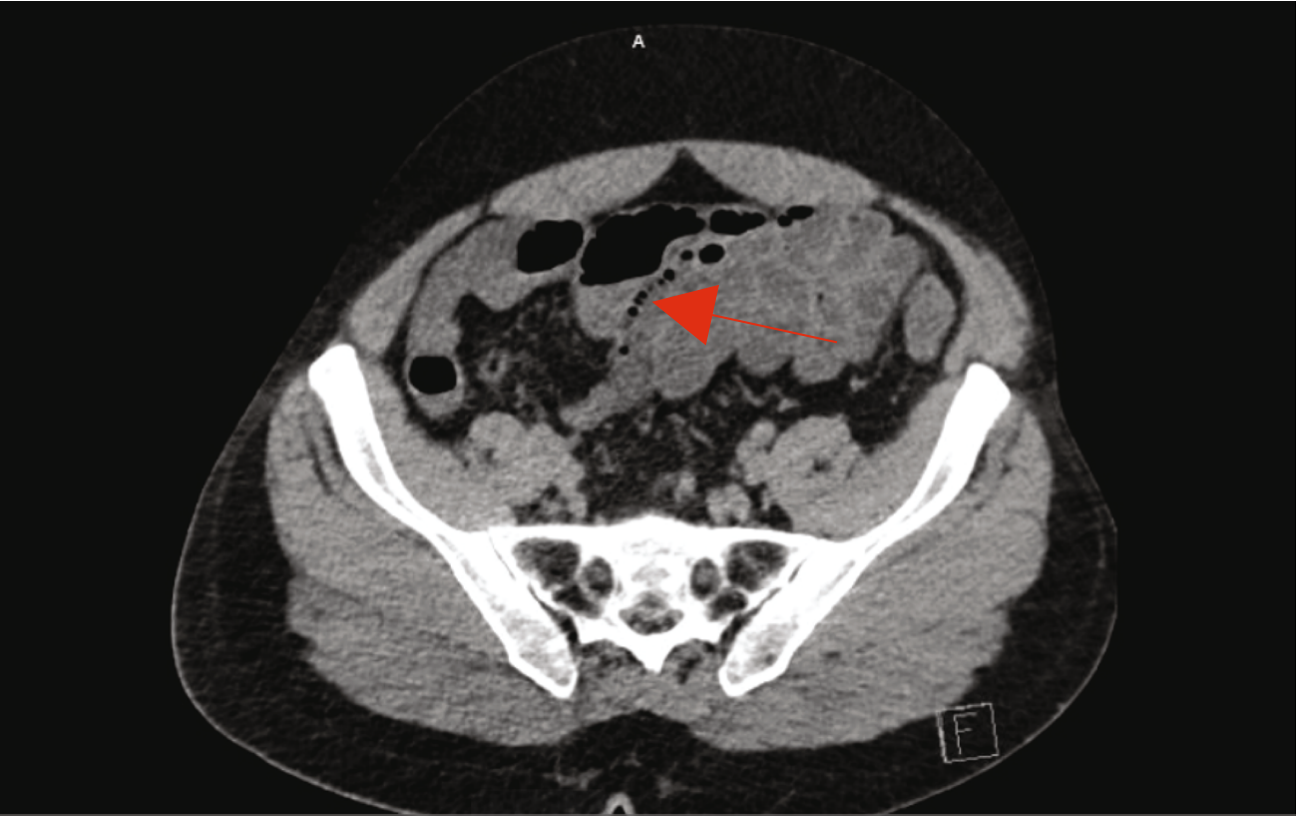
Bowel wall thickening with pneumatosis.

Radiologic workup included a head CT negative for any acute intracranial abnormalities. An abdomen/pelvis CT demonstrated portal venous gas and pan-colonic bowel wall thickening with possible pneumatosis intestinalis (Figures 1 and 2). There was no evidence of free air.

He was resuscitated with intravenous fluids and started on piperacillin-tazobactam antibiotic for the treatment of sepsis. Two sets of blood cultures were negative. Physical examination performed by the general surgical service demonstrated abdominal distension without initial findings suggestive of peritonitis. A bicarbonate infusion was initiated for metabolic acidosis and acute renal failure. Serial abdominal examinations over the course of hospital day one demonstrated worsening tenderness and distension, and the patient was taken emergently to the operating room for exploratory laparotomy.

He was found to have patchy ischemia of segments of the ascending colon and small bowel with gangrenous changes of the cecum and sections of jejunum. Therefore, he underwent a right hemicolectomy with partial small bowel resection and was left in discontinuity. The patient returned to the operating room 48 hours later for reexploration laparotomy without evidence of additional ischemia. Small bowel and ileocolonic continuity were restored with stapled side-to-side anastomoses. He remained intubated and returned to the ICU for further resuscitation. Postoperatively, renal function normalized on hospital day three. He was extubated on day seven and discharged home on day 17 with no complications.

Tissue pathology revealed segmental ischemic necrosis without perforation of the terminal ileum, cecum, ascending colon, and jejunum. The serosal surface demonstrated purple-gray discoloration with patchy areas of hemorrhage and findings consistent with marked serositis.

**Figure 3:**
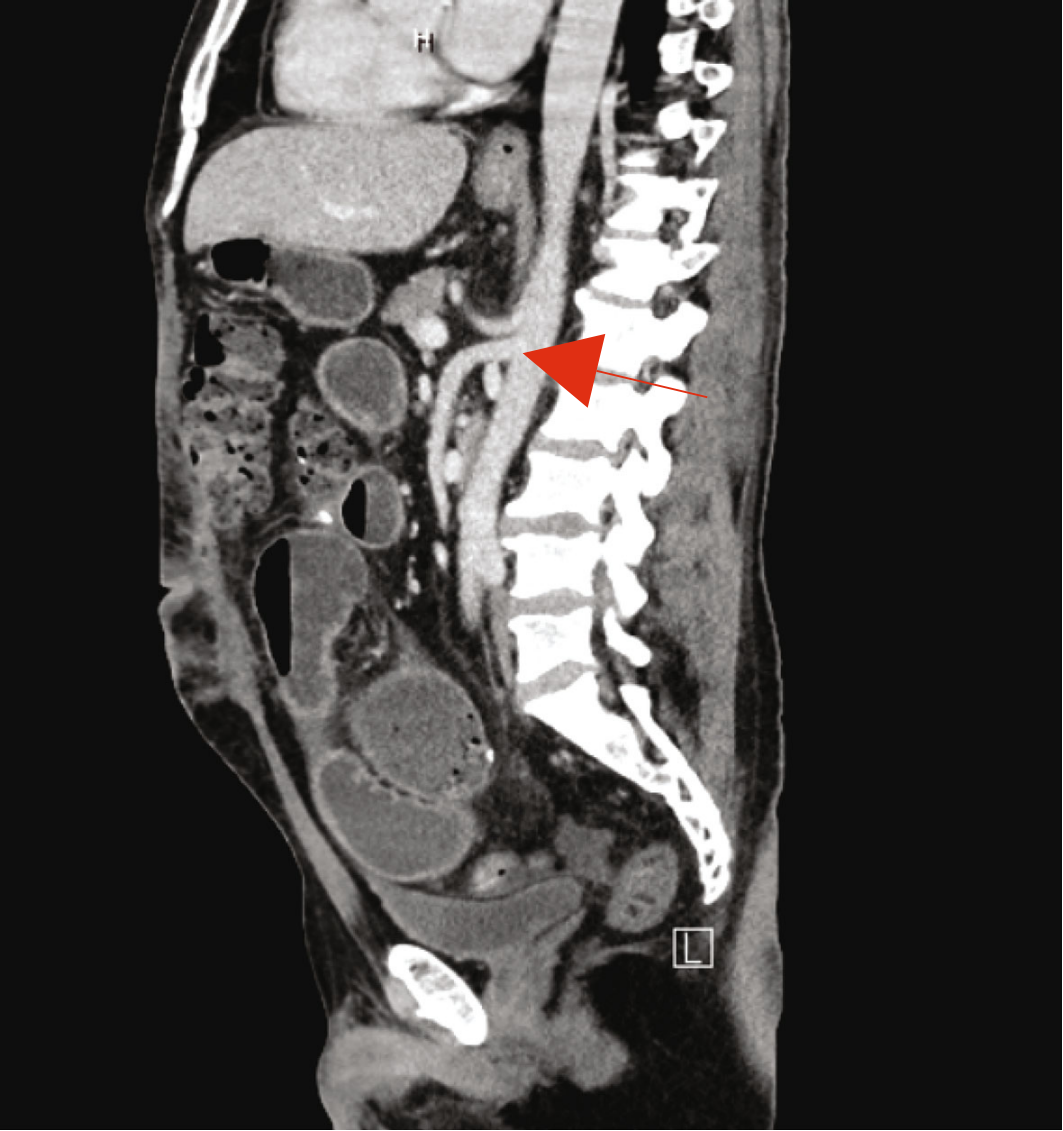
Contrast-enhanced CT demonstrating patent superior mesenteric and celiac arteries.

Additional embolic workup included CT angiography of the abdomen/pelvis that was negative for embolic foci and demonstrated patent mesenteric arteries (Figure 3).

A transthoracic echocardiogram was also negative for vegetations. *Clostridium difficile* antigen testing was negative.

## 3. Discussion

The pathophysiology of methamphetamine-induced nonocclusive bowel ischemia is likely multifactorial with a predominant effect on microvascular vasoconstriction. The sustained release of the catecholamine norepinephrine and endothelin-1 may result in vasospasm and inadequate intestinal perfusion leading to mucosal injury progressing to transmural necrosis [10, 11]. Presentation often consists of acute onset abdominal cramping and pain that may be accompanied by the development of hematochezia. Ischemic colitis is the most common in the ICU setting or patients over the age of 65 with chronic cardiovascular and/or renal disease [10]. Younger patients without significant risk factors warrant investigation of a possible drug-induced etiology, especially in areas with a high prevalence of methamphetamine abuse. Ischemic colitis has been reported in marathon runners [12].

Overall, NOMI carries a poor prognosis with a high overall mortality rate. Right-sided colonic involvement has been associated with an increased need for surgical intervention and greater mortality. Early detection of bowel ischemia is critical for patient outcomes, and computed tomography is a frequently used modality for diagnosis [13, 14]. A study by Nakamura et al. found that pneumatosis intestinalis on CT imaging may represent a harbinger of irreversible bowel ischemia necessitating early exploratory laparotomy for definitive diagnosis [15]. Supportive treatment for NOMI includes intravenous fluid resuscitation, broad-spectrum antibiotic therapy, bowel rest, and close monitoring to assess for worsening clinical status [11, 16]. Avoidance of vasoconstrictors is of vital importance as their use may exacerbate splanchnic malperfusion. Early treatment and intervention are imperative to improve survival outcomes and possibly avoid surgical resection [11, 16].

The most probable cause of bowel necrosis in our patient was likely methamphetamine-induced vascular constriction. The segmental necrosis localized to the distribution of the superior mesenteric artery is suggestive of vasospasm compared to a patchy [16]. Although the initial urine toxicology screening was negative, amphetamines have a detection window of only two to four days following ingestion [17]. The patient had subsequent positive amphetamine screens during his lengthy postsurgical clinical course and self-reported history of methamphetamine abuse prior to initial presentation.

## 4. Conclusion

Our case described a 50-year-old man with a history of methamphetamine use who presented with radiologic and pathologic findings suggestive of non-occlusive intestinal ischemia. Clinical suspicion accompanied with early diagnostic imaging and prompt aggressive treatment are key components to improving survival.

## Data Availability

Data are available on request.

## Abbreviations

NOMI: Nonocclusive mesenteric ischemia
CT: Computed tomographic
MA: Methamphetamine
mm Hg: Millimeter of mercury
bpm: Beat per minute
*μ*/L: Cubic milliliter
mEq/L: Milliequivalents per liter
C3: Complement component 3
C4: Complement component 4
mg/dL: Milligrams per deciliter
pg/mL: Picograms per milliliter
kg/m^2^: Kilogram per square meter
BMI: Body mass index
mg: Milligram
*μ*L: Microliter.
